# Role of Cyanobacterial Exopolysaccharides in Phototrophic Biofilms and in Complex Microbial Mats

**DOI:** 10.3390/life5021218

**Published:** 2015-04-01

**Authors:** Federico Rossi, Roberto De Philippis

**Affiliations:** 1 Department of Agrifood Production and Environmental Sciences, University of Florence, Piazzale delle Cascine 24, 50144 Florence, Italy; E-Mail: f.rossi@unifi.it; 2 Institute of Ecosystem Study (ISE), National Research Council (CNR), Via Madonna del Piano 10, 50019 Sesto Fiorentino (FI), Italy

**Keywords:** cyanobacteria, exopolysaccharides, biofilms, biological soil crusts, lithic surfaces

## Abstract

Exopolysaccharides (EPSs) are an important class of biopolymers with great ecological importance. In natural environments, they are a common feature of microbial biofilms, where they play key protective and structural roles. As the primary colonizers of constrained environments, such as desert soils and lithic and exposed substrates, cyanobacteria are the first contributors to the synthesis of the EPSs constituting the extracellular polymeric matrix that favors the formation of microbial associations with varying levels of complexity called biofilms. Cyanobacterial colonization represents the first step for the formation of biofilms with different levels of complexity. In all of the possible systems in which cyanobacteria are involved, the synthesis of EPSs contributes a structurally-stable and hydrated microenvironment, as well as chemical/physical protection against biotic and abiotic stress factors. Notwithstanding the important roles of cyanobacterial EPSs, many aspects related to their roles and the relative elicited biotic and abiotic factors have still to be clarified. The aim of this survey is to outline the state-of-the-art of the importance of the cyanobacterial EPS excretion, both for the producing cells and for the microbial associations in which cyanobacteria are a key component.

## 1. Introduction

Cyanobacteria are cosmopolitan prokaryotic microorganisms that can be found in a wide array of habitats, from marine to fresh waters, from soil to rocks, dwelling in temperate and extreme climates. Due to their low nutrient requirements and their high adaptability to environmental conditions, some have long been known to grow at high latitudes, characteristic of what was defined as “astonishing” [1], at temperatures exceeding 40 °C (which is the highest temperature tolerated by diatoms living in hot springs [1]) and in hyper saline environments [2]. Although 35 °C is the optimal temperature for growth, some cyanobacterial species were observed at temperatures as high as 85 °C [1].

During their life cycle, cyanobacteria exocellularly secrete outer investments mostly constituted by heteropolysaccharides, which are frequently associated with small amounts of non-carbohydrate substituents, such as peptides, DNA and fatty acids [3,4]. These exopolysaccharidic secretions (exopolysaccharides (EPSs)) may constitute up to 60% of the dry biomass (as in the case of *Nostoc commune*) [5], and their presence is considered tightly related to the capability of these organisms to successfully cope with environmental constraints and with the formation of complex microbial mats on a great variety of substrates. Notwithstanding their physiological and ecological importance, the synthesis of cyanobacterial EPSs remains to this day a complex, minimally-understood process, and a very limited amount of information on the genetics related to polymer assembly and excretion is available [6]. In addition, it is known that EPS synthesis is affected by nutritional and environmental parameters to a species-specific level [4].

Due to the massive and, in some cases, hyper-production, and due to the rheological features of these polymers, cyanobacterial EPSs have gained increasing scientific attention due to their possible biotechnological applications. Some cyanobacterial strains have had patents issued [7], although none of the EPS-derived products is currently available on the market [3]. The choice of cyanobacteria for massive EPS industrial production would benefit from their versatility, the already mentioned low nutrient requirements and the ease of the manipulation of the culture conditions in order to improve the yields by controlling the growth conditions (see Section 1.2).

On the other hand, the ecological significance of EPS excretion has been investigated, considering that cyanobacteria are among the main contributors to the constitution of the exocellular polymeric matrix (EPM) in various types of microbial biolayers. In natural environments, these complex macromolecular matrices are under constant rearrangement through the demolition and new synthesis of their constituents, in a dynamic process depending on the activity of the microbial community residing in the matrix. EPM plays a number of important roles, although some have to be demonstrated. EPSs have a putative physical protective role against several harmful factors, both chemical and physical, representing a boundary between cells and the immediate outer environment, and preserve the cells from antibacterial agents and protozoan predation [4].

More in general, the presence of a protective outermost structure seems to extend the time available for the physiological adaptation to external changes.

It has been reported that in the soil system, EPM contributes to biofilm structural stability, adhesion to the substrate, nutrient and metal ion uptake and the provision of moisture for the constitution of an optimal microenvironment [8]. In addition, EPSs seem implicated in the motility of cyanobacteria (see Section 2.4), which is important for photoacclimation. For example, in aquatic environments, cyanobacterial cells are able to reach the photic zone of the sediments near the surface after sediment mixing or sediment deposition [2].

More in general, it is thought that EPS excretion represents a physiological response to fluctuations in environmental conditions, allowing cyanobacteria to maintain their fitness and, at the same time, also sustaining the growth of other cohabiting species. Studies on cyanobacteria and on *Pseudomonas putida* showed that environmental stressors, such as the presence of heavy metals, affect not only the amount of produced EPSs, but can also affect the chemical and physical characteristics of the secreted polymer [9,10], in this way positively influencing the adaptation capability of the cells to harsh environmental conditions.

This review discusses the recent achievements in understanding the functions and the ecological role of cyanobacterial EPSs, with a specific focus on complex microbial assemblages.

### 1.1. Cyanobacterial EPSs: Chemical and Physical Characteristics

**Figure 1:**
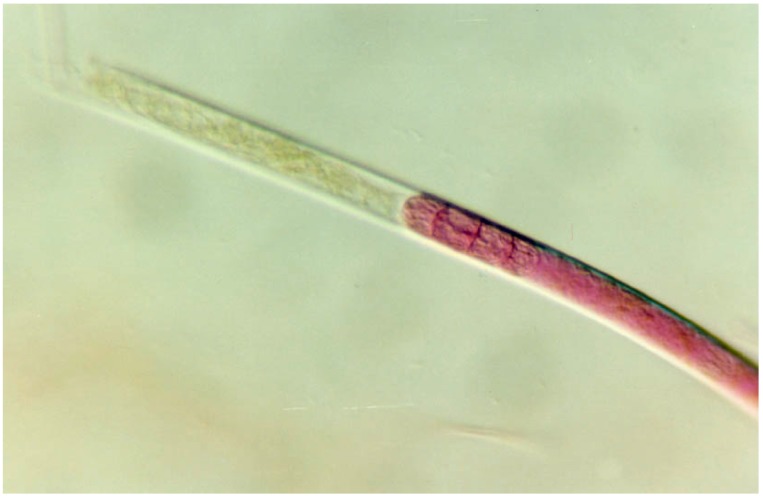
Sheath surrounding *Phormidium* sp. filaments (1000×) (Courtesy Dr. Claudio Sili, ISE-CNR, Italy).

Cyanobacteria produce mainly high molecular weight (MW) heteropolymers of a polysaccharidic nature. These secretions embedding the cells have different jellification states, according to their chemical features, but also to abiotic parameters (e.g., pH, available ions, *etc.*). More condensed fractions are usually encompassed under the terms of sheaths and capsules (capsular polysaccharides (CPSs)), which surround cells or cell groups, and are distinguished according to their thickness and consistency [4]. CPSs and sheaths reflect the shape of the cells, and while the sheath is a thin layer surrounding cells or cell groups (Figure 1), CPSs constitute a thick layer with sharp outlines, which exclude particles [4] and, thus, can be negatively-stained by using India ink.

When referring to discrete EPS particles in marine environments, the term “transparent exopolymeric particles” (TEP) was also used by some authors [11].

Less condensed EPS fractions, loosely bound to cells, are usually encompassed under the term “slime”. The slime does not reflect the shape of the cells and may include groups of cells or filaments. In all three cases, the polysaccharidic external layers can be partly released in the surrounding medium, representing the so-called released polysaccharides (RPSs). According to some evidence, CPSs and RPSs might be synthesized through different biosynthetic pathways [12], although the matter is still under debate. In addition, CPSs and RPSs may have, or not have, the same monosaccharidic composition [13,14]. In batch cultures, the amount of RPSs usually increases along with cell growth, with the consequent increase of the medium viscosity.

**Figure 2:**
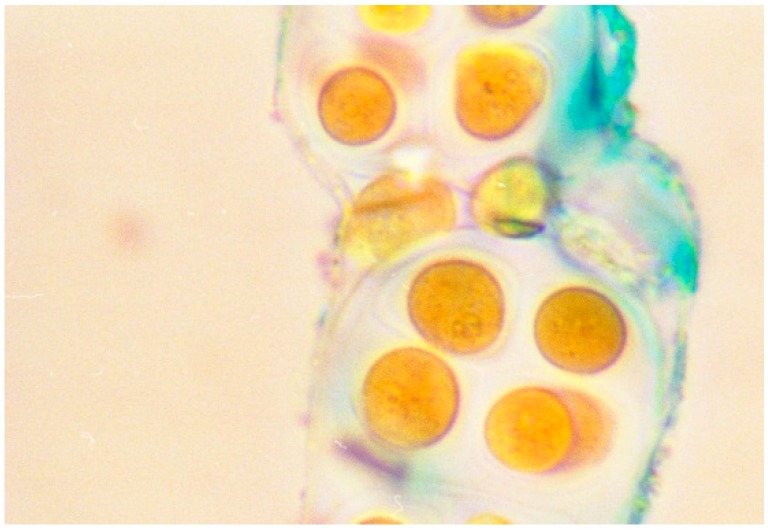
Microphotographs of *Microcystis* sp stained with Alcian blue dye to evidence the negatively-charged sheaths and slime. Scale bars = 10 µm.

Generally speaking, it is possible to stain the outermost polysaccharidic structures by using cationic dyes (e.g., Alcian blue; Figure 2), which bind to the negatively-charged groups present in the polymers.

Although other microbial groups can synthesize EPSs, cyanobacterial exudates own some peculiarities. One is the presence of sulfate groups (a feature shared with some Eukaryotes and Archaea, but not with other prokaryotes) and of uronic acids, two constituents that confer an anionic and sticky character to the macromolecules. Another peculiarity is the presence of pentoses (xylose, arabinose and ribose), which are not commonly found in the EPSs of other prokaryotes. Thanks to their negatively-charged surface, cyanobacterial EPSs usually show a high affinity for metal cations and other positively-charged or polar molecules [4,15]. On the other hand, the presence of deoxy-sugars (rhamnose and fucose), peptides and ester-linked acetyl groups can confer a contemporary hydrophobic character, significantly affecting their rheological and emulsifying properties [16], as well as the capability to adhere to solid surfaces (see Section 2.1).

About 75% of the over 160 cyanobacterial EPSs so far analyzed contain six or more different types of monosaccharides, whereas in the EPSs produced by other bacterial groups, they amount to a maximum of four [4]. However, it has to be stressed that the different extraction procedures applied for recovering the exocellular material along with different analytical approaches make it difficult to properly compare these results.

So far, more than thirteen different kinds of monosaccharides were reported for cyanobacterial EPSs [17].

The monosaccharides most frequently found in cyanobacterial EPSs are fucose, rhamnose, arabinose, galactose, glucose, mannose, xylose, galacturonic acid and glucuronic acid. Additionally, also the presence of galactosamine, glucosamine, ribose, fructose and, in some cases, also of sugars such as *N*-acetyl glucosamine, 2,3-*O*-methyl rhamnose, 3-*O*-methyl rhamnose, 4-*O*-methyl rhamnose and 3-*O*-methyl glucose [18], was reported. Generally, galactose and, above all, glucose (in more than 90% of the polymers) are major components, although in some cases, other monosaccharides (namely arabinose, xylose, galactose or fucose) may be present in higher relative amounts than glucose [3,19]. *Microcystis wesenbergii* represents a peculiar case, producing a polymer constituted exclusively by uronic acids [20].

Monosaccharides are organized in repeating units to form very complex structures. For example, repeating units of 15 sugars are reported for the RPSs synthesized by *Spirulina platensis* and *Mastigocladus laminosus* [21,22], while repeating units of eight sugars were proposed for the RPSs synthesized by *Cyanospira capsulata* [23]. Although only a few EPS structures have been so far proposed, knowledge of them is necessary in order to predict their physico-chemical properties. The high MW characterizing the majority of analyzed EPSs surely contributes to their viscosity, which in some cases, is even higher than that of xanthan gum [24]. The highest registered MW so far is 2 MDa, characterizing EPSs produced by *C. capsulata*. As of 2009, about 80% of analyzed EPSs revealed an apparent MW of at least 1 MDa [3].

### 1.2. Factors Affecting EPS Synthesis in Cyanobacteria

The chemical features of the outermost polysaccharidic structures and their abundance depends on the cyanobacterial strain considered, but also on culture or environmental conditions [24]. For the same strain, EPS characteristics may differ if determined under laboratory conditions or in natural settings, in which cells may experience nutrient limitations [24,25].

The importance of different growth parameters and the presence/absence/amount of some macronutrients have been underlined, although the effects on the EPS synthetic pathway are species-specific. The control of aeration, temperature and salinity resulted in being important for the pursuit of the optimal EPS productivity, while pH, the presence of metal ions and dilution rate were reported as possible conditioners [25].

The availability/amount and possible limitations of nitrogen, phosphate, sulfate and carbon can all influence EPS production. In particular, the C/N ratio is an important parameter. Working on different *Nostoc* species, Otero and Vincenzini [26] found that a higher amount of C compared to N levels drives EPS production in order to store the excess of C. Nonetheless, a decrease in available carbon may also lead towards EPS synthesis due to the carbon concentrating mechanism (CCM), which may enable carbon accumulation from the environment, as observed for the microalga, *Chlorella* [27].

Nutrient deprivation in some cases enhanced EPS synthesis, and thus, it was proposed as a stimulatory method in order to maximize the productivity. For example, phosphorus and nitrogen starvation worked in the case of *Spirulina platensis* [28,29], while nitrogen starvation was found effective in the case of *S. maxima* [30]. Phosphorus starvation may also induce carbohydrate accumulation over protein accumulation. In the case of *S. platensis*, carbohydrate accumulation was reported to amount to about 63% of cell dry weight [29], following P starvation.

In natural environments, EPSs help cyanobacterial cells cope with constraints, both physically and chemically. As stated in Sohm *et al.* [28], the timing of the polymer release in response to variations in environmental factors might depend on the functions that the polymer has in the different species. Many different stresses increase EPS production, including dehydration and contact with heavy metals [9].

When moisture availability increases, EPS synthesis is stimulated, as well, triggering different metabolic processes [10]. Indeed, there is a tight linkage between the occurrence of rainfall, N availability and EPS excretion [29].

Whether salinity has an effect on EPS synthesis is still controversial, and the most probably effects are species-specific. A decrease in the production may follow the increase of NaCl [30], but may be also triggered by a paucity of the same compound [25]. In other cases, no effects on the productivity related to NaCl amounts in the growth medium were observed [31]. Hyper-salinity may stimulate CPS synthesis to counteract saline water stress, as observed for the brown alga, *Aureoumbra lagunensis* [32]. The presence of the CPSs may be helpful to the cells to cope with salinity, explaining the high presence of capsulated cyanobacteria in some hyper-saline habitats [2].

Similarly to salinity, it is hard to find a general pattern for the effects of the presence of metals on EPS synthesis. It is thought that the effects on EPS synthesis are metal-specific. In some cases, a shortage of Mg^2+^ and Ca^2+^ elicited production, whereas there were no effects in other cases. The increase in EPS synthesis seems to improve the resistance to toxic metals. A study carried out by Ozturk and Aslim [9] showed that *Chroococcus* and *Synechocystis* strains resistant to Cr(VI) produced higher EPS amounts compared to the Cr(VI)-sensible isolates. It is possible that a higher EPS concentration stimulated by the metal played a role in increasing its immobilization, as suggested by Pereira *et al.* [3]. However, it was recently reported that in *Cyanothece* sp. CCY 0110, the presence of heavy metals significantly affected its protein profile, in particular with regard to the proteins associated with photosynthesis, CO_2_ fixation and carbohydrate metabolism, but did not enhance the amount of RPS released by the cells [33]. Between other possible elicitors, substances, such as glyoxylate, valerate, acetate or EDTA, are also reported [3,34].

EPS synthesis may be elicited as a physiological response to UV exposure (see Section 2.3).

Some culture conditions proved to affect the composition of the secreted EPSs. This is the case of *Chroococcus* sp. H4 after coming in contact with a 10-ppm Cr(VI) solution [9] and the case of *Nostoc* if grown alternatively with or without a nitrogen source [35]. Besides affecting the excreted quantity of EPSs, the concentration of NaCl in the growth medium may influence the composition. In the presence of combined N, a higher EPS-to-microbial biomass ratio and the synthesis of a more complex EPS structure were observed for *Nostoc commune* [35].

## 2. Role of Cyanobacterial EPSs in Microbial Mats

In natural environments, cyanobacteria are present as free-floating or as surface-bound cells. Within each of these two states, significant variations may be encountered according to the physical and chemical differences of the substrate, the occurrence of dry-wet cycle and the type and extent of constraints to which cells are subjected. Consortial activities are required for many processes that are not possible for single cells. In addition, some species (e.g., microalgae, mosses), which would not be able to colonize constrained environments, benefit from the presence of cyanobacteria, typically capable of creating microenvironments characterized by low-abiotic stress conditions (e.g., higher moisture amount, higher nutrient amount, a more stable substrate). Cyanobacteria own surprising metabolic stress responses and are characterized by physiological active-dormant-active transitions, allowing them to cope with fluctuations in moisture, illumination, salinity and nutrients. For this reason, cyanobacteria-dominated biofilms, even though differing in structure and microbial composition, colonize an astonishing variety of substrates, such as desert soils, stone monuments and even hypogeal environments [36].

Cyanobacteria are often the first colonizers of coherent and incoherent bare surfaces and may give rise to very complex microbial associations, sometimes referred to as “microbial mats”, when characterized by a laminated, multilayered structure.

**Figure 3:**
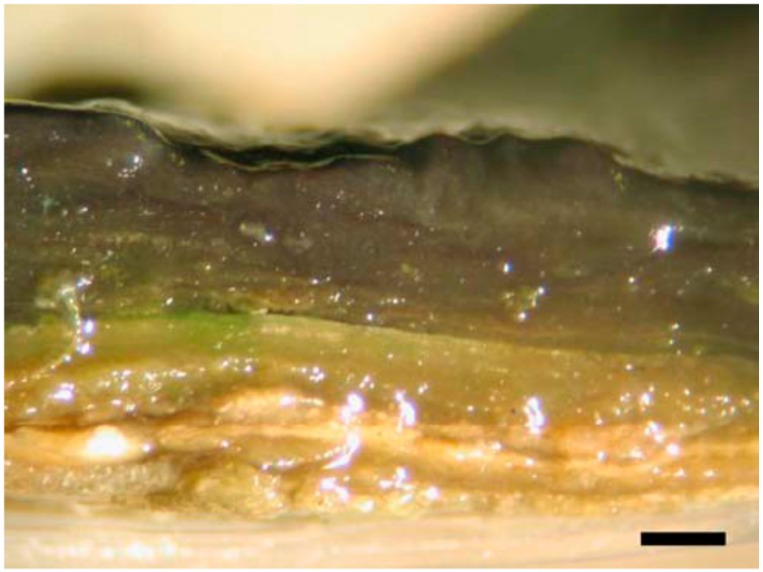
Optical microphotograph of a cross-section of a phototrophic biofilm showing the multilayered structure and the highly-hydrated EPS investment. Bar = 1 mm. Retrieved from [37] with permission.

Phototrophic biofilms and biological soil crusts (BSCs) are two examples for which the role of cyanobacterial-produced EPSs are very important for the establishment and the maintenance of the community (Table 1). In phototrophic biofilms, the surface-attached community draws its energy and carbon source almost entirely from light energy and CO_2_ fixation due to cyanobacteria, while BSCs are composed of different trophic levels and characterized by a higher community complexity (see Section 2.2). In phototrophic biofilms, cyanobacteria are usually stratified at the surface with other oxygen-evolving phototrophs (diatoms, microalgae), while green and purple sulfur bacteria occupy the lower layers, intermixing with *Chloroflexi*-like bacteria [37] (Figure 3). Phototrophic biofilms can be found growing on exposed lithic substrates, and cyanobacterial EPSs play key roles in protecting from UV irradiation and drought [38]. BSCs are composed of a phototrophic fraction in which cyanobacteria are often representative and a heterotrophic fraction, composed of microfungi and heterotrophic bacteria. In later stages, mosses, lichens and liverworts can be recruited [39].

**Table 1:** Roles of exopolysaccharides (EPSs) in phototrophic biofilms and in microbial mats.

Role	Notes	Section
Adhesion	The adhesion is determined by the hydrophobic characteristics of the EPSs	2.1
Structure	The extracellular polymeric matrix (EPM) constituted by EPS gives structure to the soil pore system and determines soil particle organization	2.2
Protection against a-biotic stresses	EPSs counteract water stress, UV stress, physical and chemical stresses	2.3
Bioweathering processes	EPSs promote mineral concentration and induce pore cracking by drying/swelling cycles	2.4
Gliding motility	EPSs excretion is involved in cell propulsion	2.5
Nutrient repositories	EPSs contribute to the concentration of nutrients; EPS constitute a C source for heterotrophic microorganisms	2.6

### 2.1. Role of EPSs in Cyanobacterial Adhesion to Solid Surfaces and Particles

The excretion of EPSs is a key step in the adhesion of cyanobacterial cells to solid surfaces, a process that plays an essential role in the formation of phototrophic biofilms and of BSCs. Indeed, CPSs seem to enhance the microbial adhesion to solid substrates and the aggregation of coarse substrates. The importance of CPSs in the adhesion capability of cells was demonstrated by comparing the behavior of mucoid and non-mucoid cells in a freshwater environment [40]. It was shown that the addition of the CPS produced by *Nostoc muscorum* to the soil was able to increase the amount of water-stable aggregates, either by gluing soil particles or by stimulating the soil community to produce more EPSs [41].

It is known that the adhesion of cyanobacterial cells to solid surfaces is enhanced by the hydrophobic characteristics of the EPSs [42], which in bacterial EPSs depend on the presence and the amount of the deoxy-hexoses, fucose and rhamnose [43], and of ester-linked acetyl groups and peptidic moieties [44]. The role of the hydrophobic nature of EPSs in the adhesion process is also supported by the observation that the excretion by *Phormidium* sp. of the sulfated polysaccharide, emulcyan, known to be capable of masking the hydrophobicity of EPSs, caused cell detachment from solid surfaces [45]. Benthic cyanobacteria *Phormidium* J-1 and *Anabaenopsis circularis* 6720, which are able to coflocculate with suspended clay particles, attach to the benthos thanks to hydrophobic interactions [46].

In biofilms and in BSCs, the initial cyanobacterial layer, tightly attached to the solid substrate thanks to the adhesive properties of the EPSs, usually grows in thickness with time, recruiting other microbial species along the developmental stages. One notable example of pioneer cyanobacteria is represented by *Microcoleus vaginatus*, a prevalent species in arid soil systems (see below). In marine environments, cyanobacterial and diatom-produced EPSs form a matrix that affords stability to mudflat sediments against erosion [47] and enriching sediments with organic matter and nutrients [48].

### 2.2. The Role of EPSs in Biological Soil Crusts

Soil colonization by *M. vaginatus* naturally represents the first stage in the formation of BSCs [49], which can represent up to 70% of the living cover in drylands [50]. The pioneering nature of *M. vaginatus* can be exploited for biotechnological applications. Artificial introduction of *M. vaginatus* on nutrient-poor/disturbed soils, an approach that can be encompassed by the so-called inoculation-based techniques, turned out to be very promising to pursue land restoration and counteract desertification [51,52]. By artificially reproducing the natural cyanobacterial colonization process, it is possible to trigger the development of induced BSCs. According to Chen *et al.* [51], the development of visible cyanobacterial crusts is very quick after inoculation, taking only a couple of weeks. Nonetheless, the constitution of the EPM in a bio-layer is a complex and longer process, which needs the whole crust community to complete. Though cyanobacteria are major EPS producers, diatoms and microalgae contribute to the biosynthetic process. When present, microfungi are notable producers, as well, and their role as a bio-layer-structuring organisms was pointed out [49,52,53]. Although the introduction of other organisms affects the final EPM characteristics, cyanobacteria remain the main EPS contributors throughout the biofilm development.

Due to the lack of UV-screening pigments, *M. vaginatus* naturally stratifies in the soil subsurface, only gliding up to the surface for short periods when the soil is moistened. On the contrary, species, such as *Scytonema* and *Nostoc*, which synthesize UV-absorbing substances, stratify directly at the soil surface [50]. A thick sheath encases bundles of *M. vaginatus*. The sheath strongly adheres to and connects sand grains.

**Figure 4:**
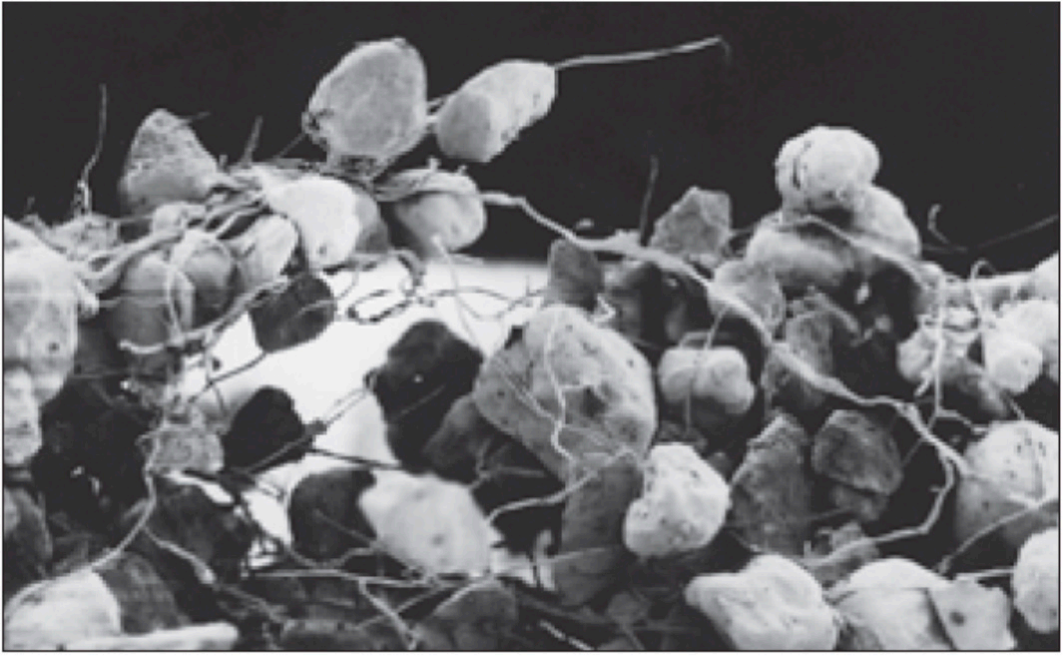
SEM images of sheathed *M. vaginatus* filaments entrapping soil particles in desert biological soil crusts. Retrieved from [50].

**Figure 5:**
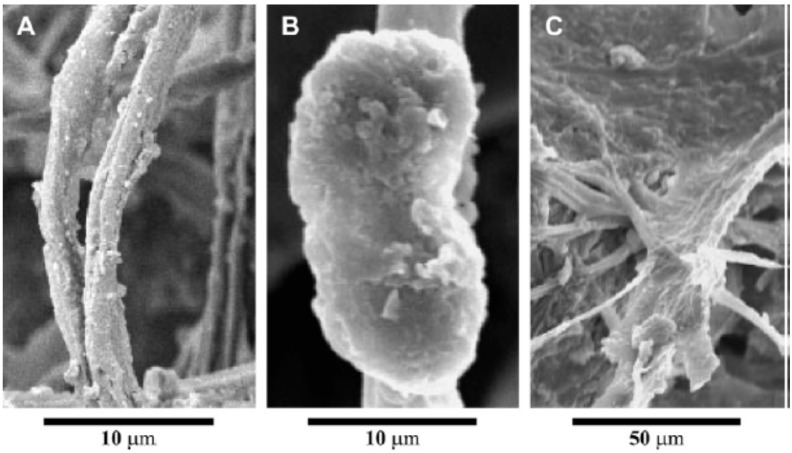
SEM images of EPSs in desert biological soil crusts: (**A**) capsule; (**B**) granules; and (**C**) slime. Retrieved from [8] with permission.

On coarse substrates, polysaccharidic exudates play a structural role together with microbial filaments, creating a cohesive net in which soil and cells remain embedded (Figure 4). Mager and Thomas [8] classify EPSs in biological soil crusts as capsules, granules and slime (Figure 5). Cyanobacterial filaments and exudates have a key role in structuring the soil pore system and soil particle organization [54]. Soil pores have an occurrence related to the physico-chemical properties of the EPSs: the higher the complexity of the surface cyanobacterial network, the greater the number of the microbial-generated pores.

**Figure 6:**
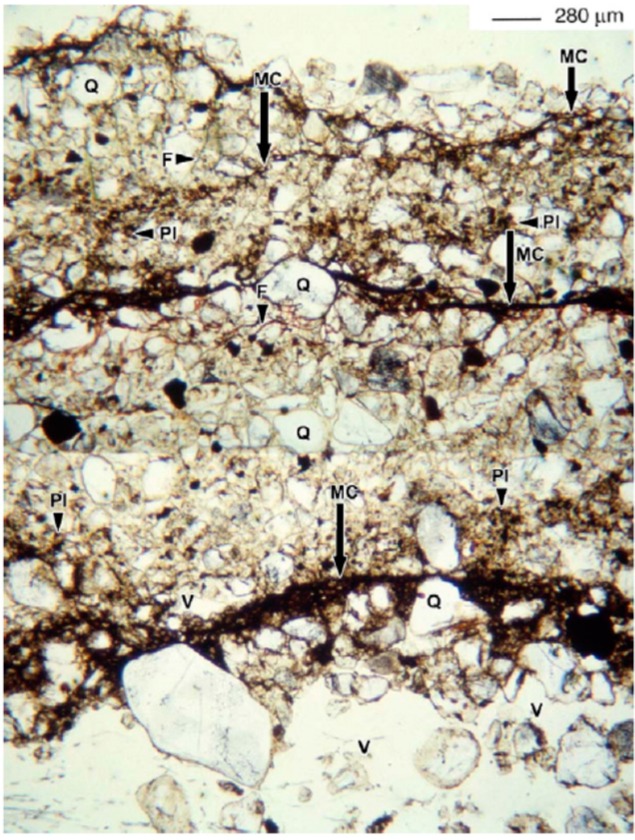
Thin section of biological soil crust from the western region of Niger showing the different layers. Q, coarse particles; PI, fine particles; MC, cyanobacterial filaments in the interspaces of the grains; F, cyanobacterial filaments; V, large pores. Retrieved from [54] with permission.

Thin sections of BSCs (Figure 6) show how cyanobacterial filaments grow in the grain interspaces. Where cyanobacteria are absent or distributed sporadically, voids between particles appear to be filled with fine particles.

In the crust layers, cyanobacteria create a continuous association with coarse and fine particles, while the sub-crust is more characterized by large pores. Possibly, the concentration of clay in the cyanobacterial-dominated layers is owed to the fact that mucous material easily binds airborne particles. Positively-charged macronutrients bind to clay and to EPSs, increasing their availability to the microflora.

Similar to what takes place in marine environments [55], in the soil system, EPSs promote spatial organization to optimize both interactions between cells [56] and nutrient and water transport [57]. Indeed, the structure dismantled through artificial EPS removal from induced BSCs significantly altered water diffusion through the soil profile [57].

### 2.3. The Role of EPSs in Tolerance to Water Stress and UV Radiation

Since excessive solar radiation and low water availability are often two factors to cope with, the protection against water stress and UV radiation is one of the main studied roles of the EPM in constrained environments.

It is a fact that cyanobacteria isolated from very dry environments, such as desert soils or the lithic surfaces of monuments, display the capacity of excreting large amounts of EPSs [37,57,58,59], a trait underlining adaptation to drought. Dehydration effects have been thoroughly studied [60]. Essentially, water stress leads to the loss of membrane structural integrity and the loss of macromolecule functioning [61], so that some authors associate cell death under drought conditions just to the loss of membrane integrity [55,56,62,63].

Although the role of EPSs in water stress has not been fully clarified, they are reportedly involved in maintaining hydration thanks to their hydrophilic/hydrophobic characteristics (see Section 1.1), which determine a gelatinous envelope around the cells that regulates water uptake and water loss processes [3]. Furthermore, they stabilize cell membranes along with non-reducing sugars sucrose and trehalose [5]. In soil systems, the hygroscopic nature of cyanobacterial sheaths becomes evident when the surface is wet, when they can be seen swelling to extensively cover the surface [64]. Cyanobacteria can absorb water many times their dry weight [65]. For example, colonies of *Nostoc* reportedly increase their mean diameter from 50–100 µm to 150–250 µm after wetting. At the same time, cyanobacterial filaments are extruded out of the sheaths, to be retracted inside when the general moisture level decreases [64]. One of the strongest pieces of evidence for the role of EPSs in the water stress tolerance was provided by *N. commune* by Tamaru *et al.* [66]. EPS-deprived cells were significantly harmed in their capability to evolve O_2_, and a decrease in cell viability was observed. In addition, EPSs are also thought to confer an increase in freeze tolerance, although the related involved mechanisms are scarcely known [61].

Cyanobacterial EPSs are also beneficial to neighboring cells in microbial associations. EPSs provide for the structuring of the biofilms, creating preferential flows of water and nutrients. In addition, EPM creates hydrated microenvironments in which the cells are protected from harmful solar radiation and physical harm and represent a source of carbon for heterotrophs. Under laboratory conditions, Knowles and Castenholz proved that EPSs produced by *Nostoc* sp. CCMEE 6160 improved water stress tolerance of the naturally co-habiting microalga, *Chlorella* sp. CCMEE 6038, which does not produce EPSs [61]. Although the lab conditions probably modeled the corresponding real environmental conditions only to some extent, this study is supportive of the hypothesis.

In BSCs, EPSs are involved in water capture from rainfall and non-rainfall sources, so that water content is higher in crust-covered soils compared to bare neighboring counterparts. The abundance of EPSs was proven to be positively correlated with the water capture capability of the biological crusts. In addition, a significant difference in water-retaining capability after treating soil crust samples for EPM removal was detected [53]. Following a significant water introduction, the swelling of the EPSs is reported to cause soil pore clogging, possibly leading to water run-off [54]. On the other hand, recent findings point out that the presence of EPSs in biological soil crusts is key in conferring a “spongy” structure to the substrate, suggesting a more complex role of the EPM in optimizing water distribution through the soil profile [57].

EPSs intervene in preserving the stability of the membrane vesicles during cycles of drying and swelling [5,67], as well as stabilizing desiccation-related enzymes and molecules [68]. As an example, the addition *in vitro* of the EPSs of *Nostoc commune* CHEN to membrane vesicles prevented them from fusing, counteracting one of the unwanted outcomes of the rehydration process.

The synthesis of trehalose by some cyanobacterial strains is in concert with EPS synthesis, which can occur in response to water loss during desiccation [69]. Although this does not seem to help cope with water stress in the case of *Nostoc verrucosum* [66], trehalose is thought to intervene in preserving cell membranes during water stress in other instances [69]. Trehalose is obtained by the transformation of the α (1,4)-glucose polymers into α (1,1)- by the maltooligosyl trehalose synthase, replacing hydrating water molecules, producing vitrification [70]

**Figure 7:**
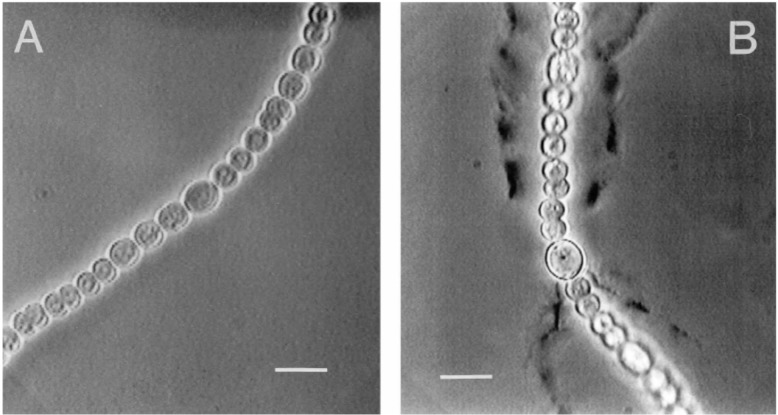
(**A**) Microphotograph of *Nostoc commune* DRH1 not exposed to UV; (**B**) *N. commune* after 72 h of exposure to UVB. Bars = 10 µm. Retrieved from [71] with permission.

Long UV-B exposures of *Nostoc commune* DRH1 resulted in higher CPSs excretion at the cost of reduced cell number (Figure 7).

The thicker the capsular envelope, the longer the path length of radiation to reach the cells. In addition, exocellular polysaccharidic structures may contain UV-absorbing compounds, such as scytonemin [72], which was found in the glycan matrix of *Nostoc commune* [73] and mycosporine amino acid (MAA)-like substances [74], which contribute to an absorption maxima between 310 and 360 nm, and at 370 nm, respectively. The synthesis of these pigments is elicited by UV exposure, as well [38,71].

### 2.4. Role of EPSs in Bio-Weathering Processes

**Figure 8:**
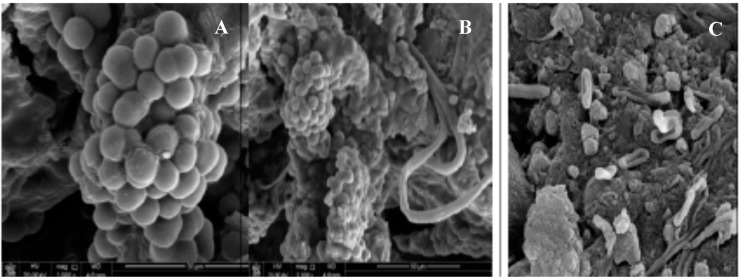
SEM microphotographs of rock-dwelling phototrophic biofilm. (**A**) Sheathed *Chroococcidiopsis* cells; (**B**) unicellular and filamentous cyanobacteria; and (**C**) heterotrophic bacteria. Retrieved from [80] with permission.

The excretion of EPSs is also key in lithic substrate colonization by epilithic and endolithic cyanobacteria and in the following bio-weathering processes (Figure 8). The colonization of sub-surface niches helps to counteract UV, thermal and water stress, which are greater for surface-dwelling communities. The capability to alter stone surfaces is owed to their capability to adhere and penetrate within the rock pore spaces, causing the exfoliation of the upper substrate layers, as well as causing irreversible unaesthetic staining due to the release of pigments. Several investigations aimed at defining the role of EPSs in the fouling caused by cyanobacterial colonization of stone artwork, in order to elaborate potential control strategies [35,36].

About 20%–30% of stone deterioration has reportedly a biological origin [75]. Stone weathering is carried out by microorganisms by penetrating and pushing apart the cracks in the mineral substrate through cycles of drying/swelling and warming/cooling. By swelling when wetted, the mucous secretions exert a great pressure from within. At the same time, mineral dissolution takes place following the release of acidic compounds, Ca^2+^, OH^−^ and organic ligands [76].

In the first rock layers, EPM can concentrate metal cations and nutrients present at low concentrations, sequestering them directly from the substrate. Welch and Vandevivere [77] showed how microbial EPSs enhance the dissolution of feldspathic substrates, while forming complexes with framework ions in solution. Metal cations, such as Ca^2+^, Mg^2+^ and Fe^2+^, are important for cell metabolism and for the stabilization of the structure of biofilms. Indeed, in biofilms, electrostatic interactions produced by cations provide cohesion [78]. Cations serve both as cross-linkers in the biofilm matrix and stimulate physiology-dependent attachment processes in microbial cells by acting as cellular cations andenzyme cofactors [79].

In a recent study, *Plectonema*, *Gloeocapsa*, *Gloeocapsosis* and *Leptolyngbya* strains isolated from epilithic biofilms showed a good affinity for Ca^2+^, Mg^2+^ and Fe^2+^, although to different extents. The interactions of cells-metals appeared mainly driven by ionic forces, the specific amount of Ca^2+^ and Mg^2+^ removed by the cyanobacteria (*i.e.*, metal removed per gram of cell dry weight) being somewhat correlated to the anionic density of the capsulated or sheathed cells [38]. Divalent cations Ca^2+^ and Mg^2+^ form cross-bridges with the charged fractions of the exopolysaccharidic strands, increasing the cohesion of the secretions. In fact, the use of complexing agents, such as EDTA and EGTA, or the use of Dowex resins cause the EPM to fall apart. EDTA solution was successfully used to remove EPSs from coarse and sandy substrates [36].

Additionally, the capability of selectively-immobilize toxic heavy metals [12,81] could represent a defensive strategy to prevent them from reaching the cells.

### 2.5. Role of EPSs in Cyanobacterial Gliding Motility

The proposal of the role of EPSs in gliding motility dates back to the 1920s [82]. It is a fact that cyanobacteria secrete slime while gliding. Hoiczyk and Baumeister [83], who observed that EPSs are extruded through junctional pore complexes (JPCs), fourthly supported the theory. JPCs are prokaryotic organelles with diameters ranging from 70 to 80 nm and 32 nm long, spanning the cell wall. A linked channel, 13 nm in diameter, spans the peptidoglycan layer. In *Phormidium uncinatum* and *Anabaena variabilis*, JPCs are located near the cell septa, at angles of 30–40°, related to the cell axis. The slime extrusion likely propels the cell forward [84]. Oscillin, a Ca^2+^-containing protein on the surface of *Phormidium* sp., possibly determines that channels that direct the EPS flow. If oscillin is arranged elliptically, the cell will rotate; if the filaments are arranged radially, the cell will not rotate [85]. Currently, the mechanisms leading to the propulsion are not clear, and some open questions remain. For example, it is unclear if a difference in the chemical characteristics of EPSs may influence the motility.

### 2.6. Cyanobacterial EPSs as a C-Source

The composition of the producing community fraction, environmental conditions (see Section 1.2) and biochemical processes at the community level influence the chemical and physical characteristics of the EPM. In oligotrophic conditions, EPSs represent a notable source of organic C available for cross-feeding processes. By these means, the activity of the producing organisms is balanced by the activity of the consumers, whereas C from EPSs is the primary substrate respired after rainfall events in deserts [86]. Contrary to what one would expect [8,34], under nutrient limitations, the cyanobacteria-contributed EPM in constrained conditions may result in being quantitatively and qualitatively complex [36,48]. The macromolecule may contain a large number of sugar constituents (up to 13), including pentoses, hexoses, amino-sugars and carboxylic sugars [36,48], organized in polymers with different MWs [48]. The capability to degrade these chemically-complex exudates needs a wide set of enzymes produced by different organisms, recruited along the developmental stages of the biofilm. Indeed, enzymatic activity increases moving from initial cyanobacteria-dominated biolayers to complex and, especially, lichen-colonized biolayers. High MW compounds decompose to simple low MW compounds and are metabolized by heterotrophs or used as protection against desiccation [8]. With the introduction of new colonizers, the C/N ratio increases, indicating carbon inflow and the accumulation of N-poor organic remains. In a recent study, Miralles *et al.* [87] observed that the enzymatic activity within BSCs is directed towards low MW substrates. This may be interpreted in accordance with the pioneering nature of crust organisms (which tend to assimilate first simple and readily available compounds) and/or may indicate the accumulation of organic matter in order to develop soil structures and proper microhabitats. This latter conclusion is supported by a recent study [88] in which sucrase and hydrolase activities were mainly directed towards less condensed (referred to as “colloidal”) EPS fractions. Due to the enzymatic activity, higher amounts of low MW colloidal EPSs were present in younger BSCs, whereas more condensed and relatively insoluble EPS fractions were preserved from degradation in older BSCs.

## 3. Conclusions

From the above reported experimental findings, it appears evident that the EPM plays an essential role in protecting from harmful environmental factors the microbial community residing in BSCs or in biofilms. However, in spite of the large number of studies claiming this role, only a few of them directly investigated the molecular mechanisms leading to the synthesis and release of EPSs under environmental stress conditions. Further studies in this direction are needed in order to clarify the way in which environmental factors activate the biosynthetic machinery leading to the synthesis of the EPSs and to explain the differences observed in the reaction of EPS-producing cyanobacteria to stresses of the same nature.

Another topic that has been almost completely neglected until now is related to the understanding of the possible differences in the protective functions of EPSs having different compositions or molecular sizes. Indeed, it is conceivable that polysaccharides with different molecular dimensions and structure or composed of monosaccharides with specific properties (e.g., hydrophobicity or hydrophilicity; negative charge, *etc.*) protect to different extents the cells from harmful environmental conditions. Therefore, the cyanobacterium able to modulate these characteristics may have an ecological advantage and might be able to better thrive in harsh environments.

Finally, a very important issue that has still to be investigated is the interplay between the various microorganisms residing in biofilms and in BSCs in the production and consumption of the EPM of such complex structures. Understanding the mechanisms of formation and degradation of EPM in biofilms and BSCs might lead to the capability of contrasting or favoring their formation, depending of the negative or positive role that they play in the environment in which they flourish.
